# Measuring Photonics in Photosynthesis: Combined Micro-Fourier Image Spectroscopy and Pulse Amplitude Modulated Chlorophyll Fluorimetry at the Micrometre-Scale

**DOI:** 10.3390/biomimetics7030107

**Published:** 2022-08-07

**Authors:** William P. Wardley, Johannes W. Goessling, Martin Lopez-Garcia

**Affiliations:** 1 Department of Physics, University of Exeter, Exeter EX4 4QL, UK; 2 Department of Biology and CESAM, University of Aveiro, 3810-193 Aveiro, Portugal; 3 INL—International Iberian Nanotechnology Laboratory, 4715-330 Braga, Portugal

**Keywords:** natural photonics, *Begonia* sp., diatoms, spectroscopy, chlorophyll, PAM fluorimetry

## Abstract

Natural photonic structures are common across the biological kingdoms, serving a diversity of functionalities. The study of implications of photonic structures in plants and other phototrophic organisms is still hampered by missing methodologies for determining in situ photonic properties, particularly in the context of constantly adapting photosynthetic systems controlled by acclimation mechanisms on the cellular scale. We describe an innovative approach to determining spatial and spectral photonic properties and photosynthesis activity, employing micro-Fourier Image Spectroscopy and Pulse Amplitude Modulated Chlorophyll Fluorimetry in a combined microscope setup. Using two examples from the photosynthetic realm, the dynamic Bragg-stack-like thylakoid structures of *Begonia* sp. and complex 2.5 D photonic crystal slabs from the diatom *Coscinodiscus granii*, we demonstrate how the setup can be used for measuring self-adapting photonic-photosynthetic systems and photonic properties on single-cell scales. We suggest that the setup is well-suited for the determination of photonic–photosynthetic systems in a diversity of organisms, facilitating the cellular, temporal, spectral and angular resolution of both light distribution and combined chlorophyll fluorescence determination. As the catalogue of photonic structure from photosynthetic organisms is rich and diverse in examples, a deepened study could inspire the design of novel optical- and light-harvesting technologies.

## 1. Introduction

Natural photonics, the study of light–matter interactions in nanostructured biological systems, is a growing and diversifying field. Research has focused on the fundamentals underlying those natural photonic systems, as well as on their implications in nature and how to utilize them as inspiration in biomimicry. There is a vast amount of work on understanding the photonic properties of nano-scale structures found in a variety of animals, spanning from insects [1,2,3,4] to shellfish [5,6], birds [7,8,9] and beyond [10,11]. Animals employ photonic nanostructures for varied behaviours, such as displaying colour in mating or as aposematism, in pattern production and vision [12]. Beyond the impressive diversity in animals, photonic structures evolved into a significant number of photosynthetic organisms. While some of those examples occur in the fruit—potentially linked to attracting birds fostering reproducibility [13,14]—photonic structures are often found in close proximity to or even within the photosynthetically active organelles. Suppose these photosynthetic systems are indeed related to efficient light absorption and photosynthesis (PS). In that case, as hypothesized in some cases, it is possible they also serve as inspiration for improving a range of optical technologies, and among those is light-energy harvesting [15].

The biological function of photonic structures in many photosynthetic organisms is widely unknown. It is often speculative due to the lack of methodologies that allow measurement of the dynamic nature of photosynthesis, where light absorption and energy transfer constantly adapt to environmental triggers for an optimized process [16]. Different mechanisms control energy absorption either by enhancement under light-limited conditions or by reduction of illumination to avoid excessive energy, including structural adaptions on multiple scales from canopy modification and leaf orientation, to chloroplast movement and modulating of thylakoid membrane stacking and composition [17]. As one of the most prominent processes on Earth, photosynthesis is the primary source of biomass accumulation. It has two components: a light-driven part (the primary reaction) collects energy from sunlight, which is then turned into organic matter by the fixation of atmospheric carbon dioxide (the secondary reaction). The whole process can rapidly adapt to fluctuations in light energy and is equipped with regulatory feedback loops and energy dissipation mechanisms [18].

Photosynthesis activity can be measured at different steps along the process: Biomass accumulation and growth can be regarded as the net photosynthesis activity, where energy quenching and dissipation are not considered. Some studies determine oxygen evolution arising from water-splitting at photosystem II as a proxy for this process. Such methods are commonly performed on bulk culture samples with Clarke-type electrodes or with optical sensing techniques usable down to the cellular scale [19,20]. Another set of methods targeting the initial part of the primary reaction is based on pulse amplitude modulated (PAM) chlorophyll fluorescence measurements, employing short light pulses in the sub-second range [21]. The short pulses and low intensities facilitate the measurement of fluorescence without interfering with the photosynthetic process. This is due to temporal and intensity energy thresholds controlling the initiation of photosynthesis. Switching between dark adjustment and photosynthesis is achieved by illuminating with longer pulsed (or continuous) illumination, resulting in a detectable quenching of chlorophyll fluorescence due to energy conversion into chemical equivalents. PAM chlorophyll fluorimetric methods are powerful as they allow for non-invasive measurement and high sample throughputs but are limited by species-specific pigmentation and light-scattering structures as well as by different dissipation mechanisms in some species, hence hampering comparison between species and between different experimental designs [22].

Beyond the molecular aspects of photosynthesis, it was recently demonstrated that the thylakoid arrangement in the chloroplast might have intrinsic nanophotonic properties [23]. The stratified arrangement of the grana within the thylakoids could provide these organelles with tailored and adaptable light absorption capacity by means of specific scattering patterns. Further, thylakoid structuring can present even more sophisticated arrangements to form so-called photonic crystals [24,25], which have been suggested to provide photonic properties such as slow-light effects for enhanced light absorption. In general, it is now believed that light scattering at the intracellular level is critical for the ultimate properties of photosynthetic organisms. Further, it is possible to apply the best knowledge and practices from the study of natural photonic structures to photosynthetic structures as well. This opens the possibility of using well-known nanophotonic characterization techniques in the study of natural photosynthetic photonic organelles in vivo. If these techniques could be combined with PAM fluorescence, it will be possible to correlate photosynthetic efficiency and photonic response quantitatively in vivo and for a single organelle.

Another advanced natural photonic system has been recently described in diatoms, some of the most abundant microalgae on Earth. Diatoms form silicon dioxide shells (frustules) made of valves and girdle bands as subordinate components. Recent experiments showed that the complex 3D porous networks in the girdle band of the species *Coscinodiscus granii* open photonic bandgaps and guided modes [26], reminiscent of advanced nanostructures known as slab photonic crystals [27]. When measured in water at low refractive index in contrast to the silicon dioxide slab, the bandgap opens in the near-infrared at the normal light incidence and disperses towards the blue spectral range with an increasing angle. Simultaneously, higher-order diffraction bands occur in the green spectral range (*λ* ≈ 560 nm) when immersed in water. Between different individuals of that species, the photonic properties are highly conserved for natural refractive index conditions in water, giving rise to questions regarding potential biological functionalities, such as implications in light harvesting and photosynthesis.

The current paper will describe our recently developed methodology for measurements of local photosynthesis activity on the organelle scale using PAM techniques, combined with simultaneous determination of cellular structures' spectral light attenuation and scattering profiles. We demonstrate this methodology's wide range of application and reliability, using two diverse photosynthetic examples as model systems, both of which form photonic nanostructures within or close proximity to the photosynthetically active organelles. Furthermore, unlike many natural photonic structures in animals, photosynthetic organisms show photo-adaptive behaviours, which offer many opportunities for biomimetic design inspiration, but also some complications regarding their analysis; primarily, how to optically analyse a self-adapting system, sensitive to light application during the measurement? This paper aims to present a method that allows the analysis of both the dynamic photonic behaviour and measuring the photosynthetic characteristics of phototrophs with photonic structures.

## 2. Materials and Methods

The optical system described here relies on the combined implementation of Fourier Image Spectroscopy (FIS) and PAM, two well-established microscopy techniques. This section will describe the technical implementation of the individual optical paths necessary for each technique. Next, we will show how they are combined in the same setup to allow simultaneous FIS and PAM characterization. Finally, we evaluate specific technical problems of the combined FIS-PAM setup when compared to other systems that provide both types of measurements separately.

As different illumination sources are implemented in the setup, we defined the nomenclature used throughout the manuscript. White light, as used in the setup, refers to a halogen Tungsten lamp as the illumination source, irradiating the sample with a broad spectral range from ca. *λ* = 380 to 800 nm. Illumination from the PAM system is provided via RGB LEDs with narrow-band spectral ranges. If the LEDS are combined, the resulting illumination is referred to as LED white light. The term photosynthetic active radiation (PAR) is used when the illumination spectrum and intensities are expected to induce photochemical quenching [28].

**Figure 1:**
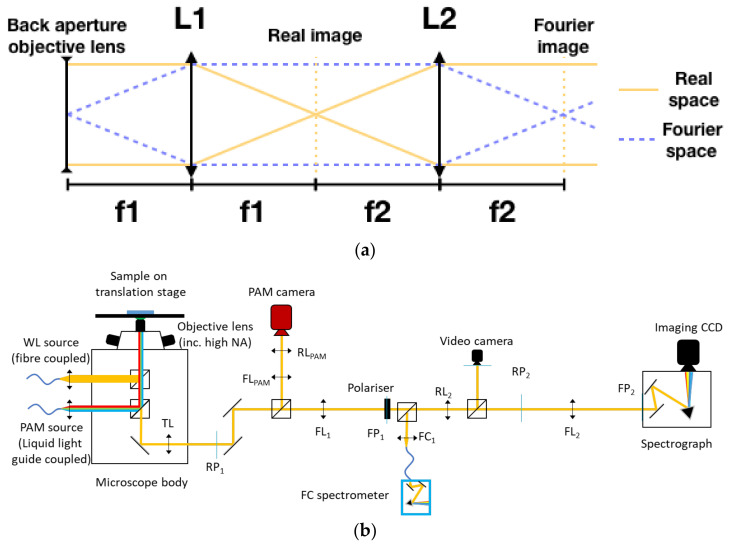
(**a**) The positioning scheme for lenses in a 4f Fourier system, with lines indicating the behaviour of both the real (imaging) and Fourier paths of light. Images are formed where the lines converge, gold for the real plane and dashed blue for the Fourier plane. This unit cell can be repeated, with the collimated beam beyond L2 being treated as that emerging from the objective lens. (**b**) Diagram of the optical system design, combining both the Fourier Image Spectroscopy and PAM detection paths. A series of the real plane (RL) and Fourier Plane (FL) lenses placed at their appropriate position, as determined by their focal lengths, guide the beam to image the Fourier plane/back aperture of the objective lens onto the spectrograph slit. The positions of the real (RP) and Fourier (FP) planes are also labelled. Distances between all lenses are the sum of their focal lengths, which can be adapted for each system based on the availability of space or resources. In this case, as the tube lens is from Nikon, f_TL_ = 200 mm, and all other lenses are 200 mm to match, except FC_1,_ which is a 5 mm lens to illuminate the fibre end.

FIS is a technique originally developed to analyse light scattering by a wide family of nanophotonic structures from plasmonics and photonic crystal structures [29]. In the most common FIS implementation, a Bertrand lens is placed between an infinite corrected objective lens and the eyepiece/imaging camera of a standard optical microscope (see Figure 1). The most common configuration is known as 4f where f is the focal length of the tube lens of the optical microscope being used [28]. The use of the extra Bertrand lens ensures that an image of the back focal plane of the objective lens is projected into the eyepiece or the imaging camera. It can be easily demonstrated using Fourier optics that the back focal plane image in an objective lens can be approximated as the optical Fourier transform of the pattern at the sample under inspection (confocal plane of the objective lens to the back focal plane) [29]. Hence, the back focal plane image shows the equivalent image of the illuminated sample but in momentum space (k-space) for the wavelengths used for illumination. Since wavevector can be easily correlated with angle, this technique allows for a fast and reliable collection of scattering patterns.

One means of obtaining these measurements is to place a sample on a rotation stage in front of a long working distance objective lens and acquire a series of measurements for different rotation angles. In that implementation, the measurement spot, of several to tens of microns, will change position as the angle is varied except at the very centre of rotation of the goniometric system, which imposes limitations on the measurement of organelles within cells, for example, as translation across the sample is no longer an option [30]. Other implementations use bulky lenses and parabolic mirrors to generate the Fourier transform of the sample image, which allows for the measurement without sample rotation. Therefore, FIS implementations with a high magnification objective lens in an optical microscope allow for both inspection of small areas (diffraction limited) and angular scanning without sample rotation. Moreover, the use of a non-rotating approach allows measurement of the response of dynamic systems, even if it varies with time, as commonly occurs in photosynthetic systems (e.g., chloroplast changing its morphology in response to high light in their environment).

Additionally, if the back focal image is projected into the slit of a spectrograph [31] or is scanned with an optical fibre connected to a spectrometer [32], it is possible to generate dispersion plots, with photon energy or frequency plotted against angle (or, proportionally, in-plane k-vector) with only very minimal mathematical data conversion of the spectrometer output files. Dispersion plotting in this way offers key advantages as far as biophotonics is concerned. Firstly, the measurement area is always kept constant since the sample and optics are not moved during the measurement, as in the goniometer-based microscatterometers mentioned above. Secondly, high magnification optical microscopy tools make it possible to measure the microscattering pattern for single organelles in-vivo and within living cells. That is, one can take advantage of the advanced optical microscopy tools to obtain scattering patterns from diffraction-limited volumes. Finally, imaging the Fourier pattern onto the slit on the front of a spectrometer makes it possible to measure the spectrally distributed dispersion plots directly. Paired with the use of an aperture to produce a small illumination area, it is possible to illuminate and collect light from single organelles, but with a large range of angles/k-vectors. The advantage of FIS in the study of natural photonics has been highlighted by the results obtained in the investigation of the reflectance of different types of intracellular natural photonic crystals, like opal-like vesicles in brown algae cells [33], so-called iridoplasts (chloroplast with photonic crystal-like thylakoid arrangements) [24] or slab photonic crystals in diatom microalgae [26].

Following the description above, to correlate the optical response of photosynthetic unicellular organisms or intracellular photosynthetic organelles, it is necessary to combine FIS and PAM to enable simultaneous measurement with both techniques. Given that PAM is a fluorescence technique it is possible to implement it in optical microscopes, and even commercial solutions [34] integrated with upright optical microscopes are available. Making use of a common optical path should then be possible to integrate FIS within the same microscopy system as the PAM. In the following, we explain the details of a possible implementation.

Illuminating samples for measuring both Fourier spectroscopy and PAM fluorescence measurements requires careful consideration. Effectively, two light sources are required to achieve reliable measurements from both systems. The fundamental requirements are that (a) the Fourier system requires a continuous spectrum source to produce reliable spectra across the wavelength range of interest and (b) the PAM measurement requires pulsed light at specific, predefined wavelengths, commonly achieved by LED illumination. In practice, the former requires a continuous white light source and the latter an array of LEDs of specific wavelengths with timing control. This means that both sources are required to produce results to a high standard; the LED white light from turning on all the PAM LEDs is not continuous, so it cannot be used for Fourier spectroscopy (LED wavelengths in Walz PAM used for this particular system are centred at 460, 510 and 630 nm; the measured spectrum of these is shown in Supplementary Figure S1). In order to produce two coincident spots from two sources, the two input ports on a microscope body are used, one for each source.

The white light used for FIS is a tungsten/halogen lamp that is coupled to an optical fibre, with the fibre-core diameter acting as an aperture and defining the illumination spot size. For the measurements described here, a 50 μm core multimode fibre was used; this can be changed if different spot sizes are required. The outcoupled light is then collimated (Olympus PLN 4× lens) and directed towards the top input port of the microscope. A polariser is placed in this beam to control the polarisation of the incoming light. This then strikes a beam splitter mounted in the microscope body and a fraction of the light is sent to the objective lens, which focuses the light onto the sample. In contrast to the FIS source, the PAM source is an array of three different coloured LEDs. By selecting either one colour of LED, or all three, either three independent wavelengths or ersatz LED white light can be produced. These are coupled to a liquid light guide (4 mm diameter), and the output is collimated and projected into the second port on the microscope and directed into the sample by a second beamsplitter.

In terms of optical alignment, switching between real and Fourier-plane imaging is a simple process in optical systems, relying on the 4f system to flip between one and the other. Figure 1a shows a set of lenses in the 4f configuration; this is a simple system for generating a Fourier plane image from an object but also includes the real imaging plane. Each subsequent lens will lie a focal length away from the focal point of the previous lens, hence the name 4f. By treating this as a unit-cell block for constructing a Fourier optical system, it is possible to construct multiple iterations to image either the real or Fourier plane onto a detector, as well as offering sites suitable for beam manipulation, such as the inclusion of polarisers or spatial or Fourier filters. By varying the focal lengths of the lenses whilst holding to the 4f basic design, it is possible to adjust the magnification of the generated image.

A schematic diagram of the microscope system is shown in Figure 1b. In this configuration, we used the body of a commercial inverted Microscope (Nikon Ti2 Eclipse). The tube lens is placed at a distance f_TL_ from the back focal plane of the objective lens by the design of the body of the microscope. A primary image is formed outside the microscope body by TL, at a distance f_TL_. An extra lens FL_1_ with f_FL1_ is added at a distance f_FL1_ from the primary real image (That is, f_TL_ + f_FL1_ from the tube lens.), which forms a 4f system formed by TL and FL_1_ that, if the focal distances are matched, will project the back focal plane image of the objective lens at a distance 4f from the sample.

In our configuration, these lenses are a Nikon objective (switchable for different magnifications) and tube lens pair mounted in a Nikon Ti2-Eclipse microscope. The imaging plane at the camera mounting position (RP_1_) is used to mount a variable iris aperture, which allows spatial filtering of the image if required. In the space between the imaging plane and FL_1_, a beam splitter is mounted to split the beam into two, with one path going to the Fourier measurement system and the other to the PAM camera. This second path will be described below. FL_1_ is confocal to the objective's back focal plane and f_FL1_ = f_TL_, generating a Fourier image at a distance of 4f from the back focal plane of the objective lens. 200 mm doublet lenses (Thorlabs L200D) are used throughout. In the system being described, it is at this point that the analyser polariser is placed, providing high polarisation quality as the beam is collimated at this point. If required, this could be moved a short distance and/or replaced with a Fourier filter to, for instance, remove a strong central maximum from a laser if one were being used. From here, the second pair of 200 mm lenses produce a Fourier image plane on the slit of a spectrograph (Princeton Instruments, Acton SpectraPro SP-2150 with attached CCD camera QImaging Retiga R6 USB3.0 Colour). Two additional side paths, which are accessed by flip-mirror mounted beamsplitters, allow complementary measurement. Firstly, a fibre-coupled spectrometer (in this case, Ocean Optics 4000+ Vis), allowing the measurement of spectra either from the entire Fourier pattern simultaneously (by use of FC_1_, a coupling lens that focuses the light onto the end facet of the fibre) or from a small region of the Fourier image by removing the lens and placing or scanning the fibre across the Fourier image. A flip mirror beamsplitter placed between lenses RL_2_ and FL_2_ allows the beam to be directed to an imaging camera (here, a Thorlabs DCC1645C CMOS camera) to allow imaging of the beam and photograph collection.

The PAM path replicates this structure, guiding the light to the PAM camera (Walz IMAGE-K6). In this case, we are interested in collecting the real image into the PAM CCD detector. Therefore, a two-lens system is used to project a magnified image form plane RP_1_ to the PAM camera. Again, lenses are placed following the 4f system design, but this time with the PAM camera placed at a real image plane.

## 3. Experimental Demonstration of Capabilities

To demonstrate the capabilities of this system design, we investigated two photosynthetic systems known for presenting well-defined natural photonic structures; (1) the diatom species *Coscinodiscus granii* bearing photonic crystal structures in the girdle band and (2) the iridoplast found in certain *Begonia* sp. forming 2D multilayer structures by dynamic thylakoid stacking.

### 3.1. FIS and PAM Combined to Study Photosynthesis in Diatoms Featuring Photonic Crystal Girdles

Previous studies have tested waveguiding in valve structures of that same species by focusing a laser beam to a few µm on a live cell [35]. Simultaneous photosynthesis measurements, employing microscopic PAM techniques, revealed that chlorophyll fluorescence quenching occurred in chloroplasts distant, non-instantly hit by the laser illumination. Fluorescence quenching and reduction of effective quantum yields were reminiscent of photosynthesis induction curves, suggesting light redistribution by guided modes and evanescent field coupling of chloroplasts to the slab. However, open questions remained due to the lack of a detailed description of the photonic system. It was thereby unclear how light could couple to the waveguide, although the laser entered beyond the critical angle. This was explained by sub-wavelengths diffraction events introduced by large valve chambers but remained purely speculative. Whether such an explanation suffices to describe the observed phenomena could be addressed by simultaneous in situ photonic and photosynthetic activity measurements.

It has been suggested that diatoms featuring photonic crystal-like structures in their frustules guide light around the cell, allowing chlorophyll on all sides of the cell to photosynthesise efficiently, even when in the shadow of the rest of the cell contents. The experiment proposed to test such a phenomenon was to illuminate a living diatom on one edge with a high magnification, tightly confined spot and to monitor the photosynthetic behaviour of chloroplasts across the cell. To ensure that the illumination was well-aligned onto the diatom girdle, the component featuring the photonic crystal structure in this species, the reflected spectrum was monitored, with the girdle band reflection presenting a strong, dispersive band corresponding to the band gap of the crystal, which prevents the transmission of light of certain wavelengths at specific angles. The silicon dioxide girdle-band structure opens highly reproducible bandgap properties at low refractive index conditions [26]. However, whether such properties could play a role in photosynthetic regulation is still debatable. Some earlier studies suggested that locally focused laser illumination onto the frustule induced photosynthesis in distant, that is not directly illuminated, chloroplasts [35]. However, these experiments were limited by missing measurements of the photonic and light scattering effects during the measurements. In the setup described in this paper, both measurements can be combined.

For the experiments presented here, the *Coscinodiscus granii* were grown following a well-documented procedure [26]. This previous work in the group had explored the photonic crystal-like behaviour of the girdle once separated from the rest of the cell and PAM measurements of *C. granii* with one edge pumped with a white light supercontinuum source had shown that the light appeared to be redistributed across the cell [36], but the two had not been combined. Here, an aliquot of diatom-containing water was pipetted onto a cover slip, and a second was placed on top to define a constant thickness of water across the whole cover slip. Using a 100× Nikon apochromatic oil immersion lens, a diatom floating horizontally relative to the microscope was located, allowing the girdle to be seen clearly, and aligned to the site of the white light from the Tungsten-halogen lamp. PAM measurements were begun without white light illumination, providing the dark adjusted data, then the fibre-coupled white light was turned on, with the spot focused onto the edge of the girdle. A 50 μm fibre was used for illumination, providing a spot size of 2 μm. This is the only PAR used during this measurement; the equivalent PAM-derived LED source is not used to induce photosynthesis. Recording the spectral response at this point allows the measurement of the photonic band gap of the girdle, confirming that the sample is well aligned and light is coupling into the frustule. PAM measurements of chloroplasts across the cell, from very near to the illumination spot to the opposite point of the cell, allow data to be collected on how light is being distributed in the scenario where light is striking the girdle.

**Figure 2:**
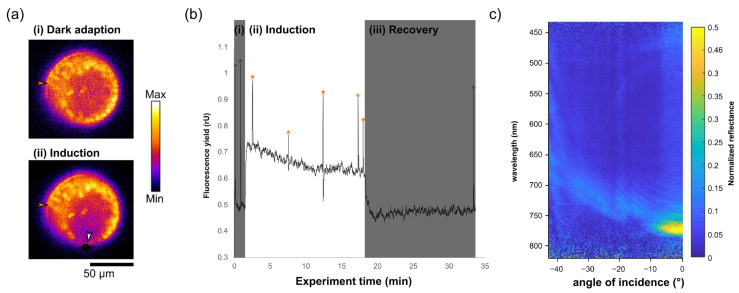
Chlorophyll fluorescence quenching and photosynthesis induction in diatoms (species *Coscinodiscus granii*). Diatoms were left in the dark for 30 min prior to the start of the experiment. (**a**) Diatom in valve-view in (i) dark-adapted state in the absence of PAR light and (ii) during photosynthesis induction when illuminated with PAR light focused to ≈5 µm spot (white arrow) onto the girdle. Chlorophyll fluorescence was recorded at low, non-PAR measuring light in a chloroplast distant to PAR illumination (orange arrow). Images are shown in false colour and altered brightness and contrast. (**b**) Graph of fluorescence yield (in relative units) versus time for the PAM excitation of the diatom. During the experiment, saturation pulses (orange asterisk above sharp peaks) were applied to estimate the oxidation potential of photosystem II. (i) shows dark adaptation (DA). Chlorophyll fluorescence rises upon illumination during (ii) the photosynthesis induction phase. Chlorophyll fluorescence decays to initial levels during (iii) recovery in the dark. Images and measurements were recorded at 100× magnification. (**c**) Angularly resolved spectral data collected using the imaging spectrograph showing the reflectance of the girdle band of a live cell in in-plane direction, showing the photonic crystal-like optical mode, the general properties of which in biological material free girdles are described elsewhere [26].

The setup allowed for the selection of single chloroplasts within a ca. 100 μm diatom cell. Furthermore, white light from a halogen tungsten lamp could be confined to a ca. 2 μm spot onto the girdle in the in-plane direction [26], Figure 2a. Upon illumination, the recorded chlorophyll fluorescence pattern resembled a typical photosynthesis induction curve [36], suggesting that light was guided inside the girdle slab and distributed to the not-directly illuminated chloroplasts. This data can be used to calculate a range of quantities to numerate the photosynthetic capabilities of cells efficiencies; here, for instance, the quantum yield of the PSII system in the dark, F_v_/F_m_, can be calculated, yielding a value of 0.54 (the calculation of which and processed data is presented in Supplementary Figure S2). When the PAR illumination was disabled, chlorophyll fluorescence recovered to initial levels (Figure 2b). Photonic properties of the girdle-band silicon dioxide slab could be measured for the first time in a living cell immersed in seawater, where a bandgap opens at λ ≈ 775 nm at normal incidence (Figure 2c). The photonic system is non-elastic due to the material properties of silica and therefore does not change. However, whether the observed effects of chlorophyll fluorescence quenching are indeed induced by photonic crystal properties, and quantification of waveguiding effects commonly occurring in the green spectral range of light in this system should be addressed in future studies.

### 3.2. Iridoplasts for FIS-PAM Test on Photosynthetic Photonic Organelles

In order to demonstrate the capacities of the FIS-PAM technique over single photosynthetic organelles, we selected the iridoplast found in *Begonia* sp. due to two key optical behaviours for this study. Firstly, when maintained in low light conditions, the iridoplasts form a multilayer Bragg-like photonic crystal structure with strong reflection in the blue [24]. The strong blue reflectance in the blue can induce slow-light phenomena in the green, as has been previously demonstrated [24]. The main consequences are an enhanced light absorption in the green spectral range, which, perhaps essentially, is the spectral range available under the canopy where the species showed live iridoplasts [25,33]. Note that this effect is opposite and counterintuitive to the photoprotective role of photonic structures in many natural systems [3]. Secondly, under exposure to higher light levels, this structure is thought to be altered by the plant, resulting in the disappearance of the strong blue reflection [37] and therefore also reducing the light absorption effect in the green. The hypothesis is that this occurs to reduce the rate of photosynthesis and prevent photodamage, so by measuring the spectral response and the photosynthetic rate via PAM for a single iridoplast, it ought to be possible to produce data to test this hypothesis, which is out of the scope of this work.

**Figure 3:**
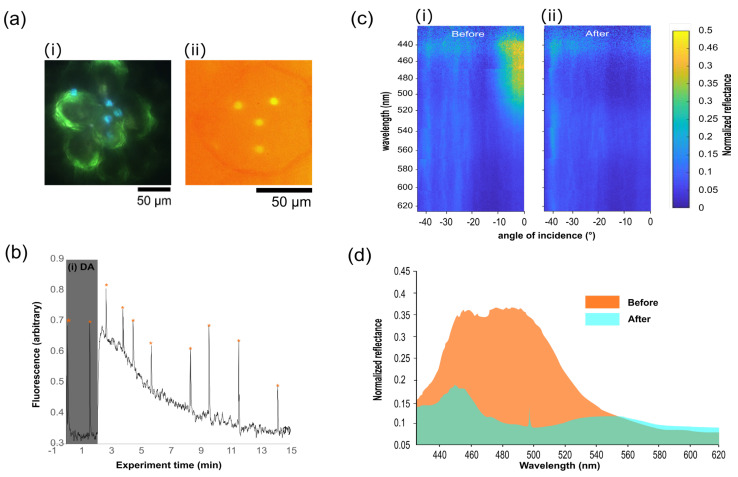
Photonic *Begonia* sp. in combined PAM-microscatterometry setup. (**a**) (i) shows sample selection in the microscope at 100× magnification. (ii) depicts fluorescence measurements of iridoplasts using the PAM camera. (**b**) PAM photosynthesis induction curve following (i) dark adaption (DA) of the sample prior to global illumination of the field of view while measuring a single iridoplast. Saturating light pulses (orange asterisk) were applied immediately before spectrograph measurements of photonic properties were performed. (**c**) Angularly resolved spectral data collected using the imaging spectrograph showing the reflectance of an iridoplast (i) before and (ii) after the PAM experiment, demonstrating the dynamic system where reflectance disappears after prolonged exposure to PAR. (**d**) Extracted spectral data from (**c**) at normal incidence before (orange) and after (green) light adaptation.

A small piece of leaf was cut from the plant (approximately 5 × 5 mm), avoiding major veins to allow the sample to lie flat on a coverslip. This leaf section was then placed upside down onto a coverslip along with a small drop of water. The focus was found on one edge of the sample using white light. Once found, the white light was blocked, and the measurement’s PAM light source was used to track the focus across the sample while not triggering photosynthesis. A new area, unexposed to white light, was chosen and focus on the iridoplast was performed using the PAM camera and pulsed light, again preventing the onset of photosynthesis. A photographed example of the cells under white light illumination, when imaged on the PAM camera, is shown in Figure 3a. Note that to obtain the image of the iridoplast under white light illumination, the iridoplast are irradiated during a brief moment (<1 s), and the original white light beam is expanded via the use of an extra lens to cover a wide-field area of the sample. 

This approach fulfils two purposes. Firstly, it reduces the number of photons per area at this stage of the preparation for measurement in order to ensure that the white light triggers no PS activity at this stage. Secondly, it allows for a wider field of view, which helps understand the sample under inspection in the future measurement area. After the image had been taken, the beam was again reduced to the original spot diameter suitable for the microscatterimetry measurements described in the first part of this manuscript. One iridoplast was selected to be illuminated with white light and was positioned at the site of the white light beam, which is known from the calibration of the system, and thus no white illumination was performed at this time. PAM measurements were started, and allowed to run for several minutes to ensure the stability of the response. After several measurements were made, the white light was turned on. There is no need for the LED illumination to be activated on the PAM system as the white light here acts as the PAR and the software performs the measurements without this setting needing to be activated. The PAM measurements are continued, with Fourier spectra being taken simultaneously from this point forward. The simultaneously measured PAM data and angle vs. wavelength dispersions are shown in Figure 3b,c, respectively.

The angle-resolved plots in Figure 3c show the spectral reflection before and after illumination. In the before plot, the characteristic V-shaped dispersion of a layered reflector, or Bragg mirror, is clearly visible, with the maximum of the curve at around 500 nm. After exposure to PAR, this behaviour has nearly completely disappeared. By looking at the spectral response for a single angle, seen in the line plot in Figure 3d, show the same behaviour, with the maximum reflection peak disappearing from a value of around 0.35 to 0.1 relative to the reference silver film used to normalise the data, with a small peak at 450 nm dropping from 0.35 to 0.175. There is a small, broad peak arising at 550 nm, representing a strong repression of reflection and a shift of the remaining reflection to the green. This matches the hypothesised behaviour of iridoplasts, which is a breaking-down of the order of the iridoplasts by the expansion of the lumen layers between the absorbing thylakoid membrane layers, both increasing the central wavelength of the reflectivity and, by reducing the ordering, reducing the efficiency of the reflectance [31]. The developed setup allowed us to inspect the same iridoplast using PAM. As shown in Figure 3c, during PAM measurements in the dark—using non-PAR measuring light and short saturating light pulses—the maximum effective quantum yields were recorded. Upon illumination, the base fluorescence signal first increased prior to fluorescence quenching caused by the reduction of membrane-soluble plastiquinones, and electron transport along the thylakoid membranes. Chlorophyll fluorescence then stabilized over time, after ca. 15 min, once the photosynthetic machinery reached an equilibrium [21,36]. The developed setup has shown a capability to simultaneously monitor both photosynthetic activity and photonic properties of the iridoplast. Simultaneous spectroscopic measurements and PAM suggest that the same dark adjusted iridoplasts strongly reflected light in the green spectral range (λ ≈ 450–500 nm) as well as higher photosynthetic quantum yield. The reflectance and photosynthetic quantum yield are reduced after adaptation to high light, which agrees with previous studies [24]. However, when illuminated with high light from the tungsten/halogen source and at steady-state photosynthetic activity, the light reflection disappears (Figure 3c,d), suggesting a loss of photonic properties and an adaptation of the thylakoid stacking mechanism. Note that previous studies suggesting similar behaviour had to be recorded in a statistical approach where PAM and photonic properties were measured in separate samples under dark or light adaptation. Here we show that by using the simultaneous PAM-FIS configuration, it is possible to obtain the evolution of both mentioned properties for an individual organelle in a continuous monitoring approach in the same sample.

## 4. Conclusions

We demonstrated an experimental system design that allows simultaneous Fourier plane spectroscopy and PAM fluorescence measurements for photosynthetic organisms. This system allows for assessing dynamic photosynthetic systems and how they adapt or adjust to changes in environmental light levels, both spectrally and for photosynthetic activity. There are many organisms where the simultaneous measurement of photosynthetic and spectral properties may provide key information about the function of photonic structures or even non-photonic, but dynamic behaviours.

We believe that the system described here presents a system that offers new insights into the subcellular and single-organelle behaviour of phototrophic organisms, including in the case of the dynamic behaviour of organelles as a response to changes in the light environment. In addition, the system design is highly compatible with standard microscopy setups distributed in most life science and physics laboratories around the world, making this technique a prime candidate for an easily implemented tool for the research in-vivo of light–matter interactions in photosynthetic organisms. This is particularly relevant in the field of natural photonics, where the strategies to achieve high photosynthetic yields via natural photonic nanostructures is a promising area to tackle important global challenges such as increasing food security by enhanced crop yield.

## Data Availability

The data presented in this study are available on request from the corresponding author.

## Supplementary Materials

The following are available online at https://www.mdpi.com/article/10.3390/biomimetics7030107/s1, Supplementary Figure S1: Spectral output of the PAM LED source. Supplementary Figure S2 Calculation of photosynthetic quantum yield in the dark (Fv/Fm).
