# Supplementary material for: Measuring Photonics in Photosynthesis: Combined Micro-Fourier Image Spectroscopy and Pulse Amplitude Modulated Chlorophyll Fluorimetry at the Micrometre-Scale
Source: Biomimetics (Basel). 2022 Aug 7;7(3):107. doi: 10.3390/biomimetics7030107 (PMC9397104; doi:10.3390/biomimetics7030107)
Supplement: Supplementary file 1 [file biomimetics-07-00107-s001.zip › biomimetics-1742778-supplementary.pdf]

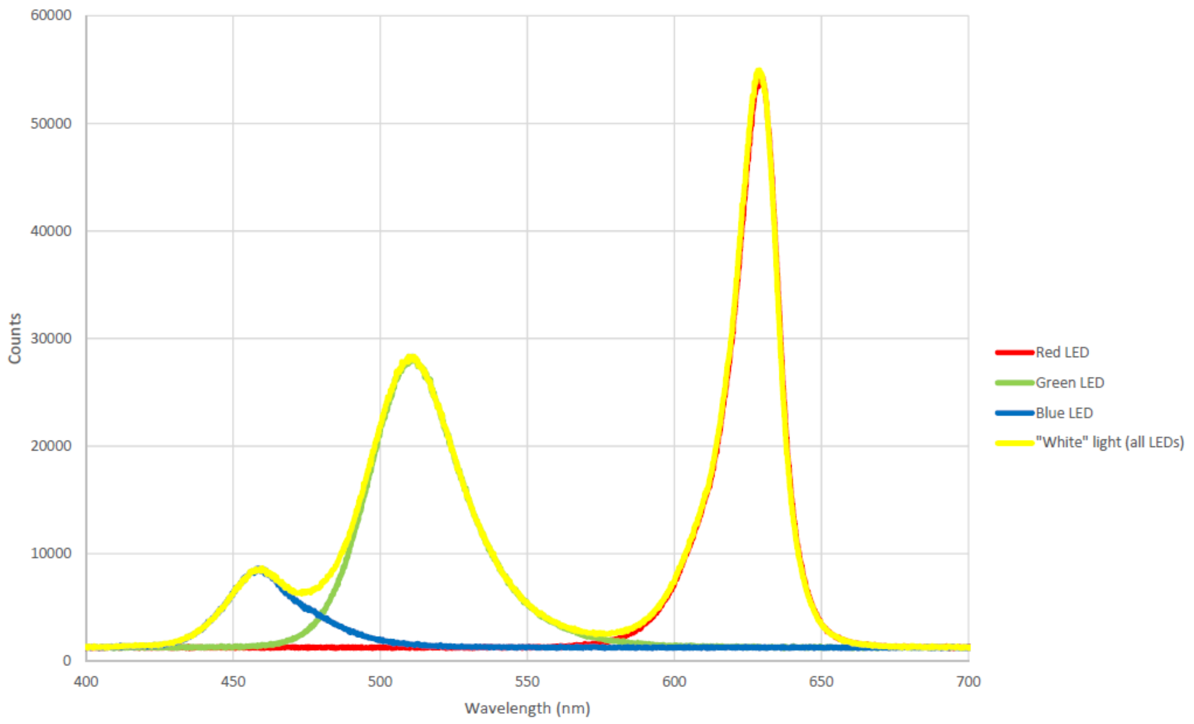

Figure S1 Graph showing the output spectrum for the PAM light source, made up of a bank of LEDs with 3 wavelengths. By running the 3 wavelengths simultaneously, "white" light can be generated

Figure S2 Calculation of photosynthetic quantum yield in the dark (Fv/Fm)

Diatom (C. granii):
F Selected chloroplast: 0.08 fluorescence counts
Fm selected chloroplast: 0.17 fluorescence counts
Fv/Fm) = (Fm-F)/Fm = 0.51

Begonia sp.:
F Selected iridoplast: 0.07 fluorescence counts
Fm selected iridoplast: 0.14 fluorescence counts
Fv/Fm) = (Fm-F)/Fm = 0.52

=(K2-J2)/K2

F	G	H	I	J	K	L
**Date**	**Time**	**min**	**min norm**	***F Selected chloroplast***	**Fm selected chloroplast**	**Y(II) selected chloroplast**
13/04/2021	17:21:40	00:00:00	0	0.08	0.17	0.51
13/04/2021	17:22:28	00:00:48	48	0.10	0.20	0.49
13/04/2021	17:24:07	00:02:27	2.27	0.16	0.12	-0.28
13/04/2021	17:29:07	00:07:27	7.27	0.17	0.21	0.20
13/04/2021	17:33:57	00:12:17	12.17	0.16	0.21	0.24
13/04/2021	17:38:52	00:17:12	17.12	0.16	0.17	0.09
13/04/2021	17:39:34	00:17:54	17.54	0.15	0.19	0.19
13/04/2021	17:54:59	00:33:19	33.19	0.11	0.21	0.47
